# Assistive Technologies, Robotics, and Automated Machines in Healthcare: Current Advances and Future Perspectives

**DOI:** 10.3390/healthcare14203447

**Published:** 2026-10-15

**Authors:** Daniele Giansanti

**Affiliations:** Centro IATIS, Istituto Superiore di Sanità, 00161 Roma, Italy; daniele.giansanti@iss.it; Tel.: +39-06-4990-2701

## 1. Assistive Technologies, Robotics, and Automation in Healthcare: An Evolving Landscape

In Assistive technologies, robotics, and automated machines are progressively reshaping the healthcare domain, supporting patients, caregivers, and healthcare professionals across an increasingly broad range of applications. Assistive technologies can facilitate independence, communication, safety, rehabilitation, and disease management, particularly among older adults and people living with chronic or disabling conditions [1]. Their adoption, however, depends not only on their potential clinical benefits but also on factors such as usability, accessibility, perceived usefulness, cost, privacy, training, and the involvement of end users in the design and implementation process [2]. In parallel, robotics is expanding its role in healthcare, particularly in physical rehabilitation, where robotic systems, including exoskeletons and other robotic-assisted devices, are increasingly being investigated to support functional recovery and patient autonomy [3]. Recent evidence suggests that robotic devices may provide beneficial effects across different rehabilitation settings, while also highlighting the importance of clinical validation, usability, cost-effectiveness and appropriate integration into existing rehabilitation pathways [4].

Automation represents another important dimension of this transformation. Automated and semi-automated systems are increasingly employed to support medication distribution and dispensing in healthcare settings, with evidence suggesting potential benefits in terms of safety, efficiency, and workflow optimization while also highlighting the need for appropriate implementation and evaluation [5,6]. Taken together, these developments illustrate a healthcare landscape in which assistive technologies, robotics, and automated machines increasingly overlap and interact, creating new possibilities for patient care and healthcare delivery.

It is within this rapidly evolving context that the Special Issue **“Assistive Technologies, Robotics and Automated Machines in the Health Domain: Third Edition”** was developed [7]. The aim was to provide a multidisciplinary forum for contributions addressing technological innovation and its applications across healthcare, encompassing assistive solutions, robotic systems, automated machines, and their integration with emerging digital and intelligent technologies. Particular relevance was given to the relationship between technological development and real-world healthcare needs, including clinical applicability, usability, safety, human–technology interaction, accessibility, and the potential impact on healthcare professionals and patients. The initiative also builds on the experience of previous editions addressing related themes, which highlighted the growing scientific interest in these technologies and their convergence within healthcare [7]. The present collection further contributes to this ongoing discussion by bringing together new perspectives and evidence on current developments, applications, challenges, and opportunities. By connecting different technological domains within a common healthcare framework, this Special Issue aims to contribute to a broader understanding of how these systems may support the evolution of patient-centered, efficient and sustainable healthcare.

## 2. Overview of Contributions

The Special Issue collected twelve scientific contributions addressing complementary aspects of assistive technologies, robotics, automation, rehabilitation, artificial intelligence and emerging digital healthcare solutions.

The six research articles (Contributions 1–6) provide perspectives on the development, evaluation and application of technology-based solutions across different healthcare and rehabilitation contexts. Contribution 1 investigated showering barriers and interaction preferences among older adults with mobility disabilities in relation to a robotic showerhead, addressing user needs and human–technology interaction in the design of assistive robotic solutions. Contribution 2 proposed an AI-driven healthcare value framework, examining the limitations of traditional healthcare value models in the context of increasingly automated and data-rich healthcare environments. Contribution 3 investigated predictive factors of inpatient rehabilitation outcomes and length of stay using machine learning, including temporal validation. Contribution 4 described the design, development and pilot usability evaluation of a multimodal conversational chatbot intended to provide emotional support and daily assistance to older adults. Contribution 5 examined the effectiveness and usefulness of assistive technology training in building workforce capacity among rehabilitation and healthcare professionals in the MENA region. Contribution 6 evaluated the usability and acceptance of three non-functional wearable garment prototypes designed for maternal health. Together, these research contributions illustrate the diversity of technological approaches being explored while addressing user interaction, usability, acceptance, professional capacity and the application of intelligent technologies within healthcare and rehabilitation settings.

The three review contributions (Contributions 7–9) address complementary dimensions of assistive technology evaluation and application. Contribution 7 critically reviewed approaches for evaluating AI-enabled assistive technologies, focusing on the transition from technical performance toward the broader concept of assistive validity and the specific challenges associated with probabilistic, data-dependent and adaptive AI-enabled systems. Contribution 8 presented a narrative review of systematic and emerging evidence concerning innovative assistive technologies for individuals with tetraplegia, bringing together developments across different technological domains. Contribution 9 provided a narrative review of assistive technologies for individuals with disabilities resulting from neurological conditions, with particular emphasis on multimodal integration. Together, these reviews extend the scope of the Special Issue from individual technological solutions toward broader questions concerning evaluation, evidence, population-specific needs and the integration of multiple assistive technologies.

Contribution 10 provides a prospective clinical research perspective through the Wear-Smile study protocol, describing a multidisciplinary telemedicine-based smoking cessation programme assisted by wearable monitoring devices and designed for evaluation through a randomized controlled trial. This contribution illustrates the incorporation of wearable monitoring into a structured healthcare intervention and its planned evaluation within a prospective clinical research framework.

Finally, Contributions 11 and 12 focus specifically on robotics and its intersection with artificial intelligence and rehabilitation. Contribution 11 systematically reviewed embodied artificial intelligence in healthcare, with particular attention to robotic perception, decision-making and clinical impact. Contribution 12 provided an overview of systematic reviews examining the therapeutic benefits of robotics and exoskeletons for gait and postural balance among children and adolescents with cerebral palsy. These contributions highlight the role of robotics as an increasingly relevant component of assistive and rehabilitation-oriented healthcare technologies.

Taken together, the twelve contributions demonstrate the multidisciplinary scope of the Special Issue and the convergence of assistive technologies, robotics, automation, artificial intelligence, wearables, telemedicine and rehabilitation. The collection ranges from user-centred investigation and usability assessment to machine learning, conversational systems, workforce training, technology evaluation frameworks, multimodal assistive solutions, clinical research protocols and evidence synthesis. Across these different perspectives, the contributions place technological development in relation to users, healthcare and rehabilitation professionals, clinical pathways and the practical contexts in which assistive and intelligent technologies are developed, evaluated and implemented.

## 3. Suggestions for Future Research and Development

The contributions collected in this Special Issue suggest several areas for further research and development. Future studies should continue to examine assistive, robotic, wearable, automated and AI-enabled technologies in relation to real-world healthcare needs, with particular attention to usability, acceptance, accessibility, human–technology interaction and the perspectives of patients and healthcare professionals. The contributions addressing AI-enabled assistive technologies and embodied artificial intelligence also indicate the importance of evaluation approaches that consider not only technical performance, but also the behaviour, interaction and practical implications of increasingly complex and adaptive systems. Further research may therefore benefit from validation across different users and healthcare settings, as well as from evaluation within existing clinical and rehabilitation pathways.

The Special Issue also illustrates the potential for combining complementary technologies, including artificial intelligence, robotics, wearables, conversational systems and telemedicine. Future research could further investigate how these technologies can be integrated while maintaining appropriate attention to safety, usability, professional training and the specific needs of different populations. In this context, multidisciplinary collaboration among technology developers, healthcare and rehabilitation professionals, researchers and end users may support the development and responsible implementation of solutions that are both technically sound and relevant to everyday healthcare practice.
